# Airborne occupational exposures and the risk of developing respiratory symptoms and airway obstruction in the Lifelines Cohort Study

**DOI:** 10.1136/thoraxjnl-2020-216721

**Published:** 2021-03-02

**Authors:** Md Omar Faruque, H Marike Boezen, Hans Kromhout, Roel Vermeulen, Ute Bültmann, Judith M Vonk

**Affiliations:** 1 University Medical Center Groningen, Department of Epidemiology, University of Groningen, Groningen, Netherlands; 2 University Medical Center Groningen, Groningen Research Institute for Asthma and COPD (GRIAC), University of Groningen, Groningen, Netherlands; 3 Institute for Risk Assessment Sciences (IRAS), Division of Environmental Epidemiology, Utrecht University, Utrecht, Netherlands; 4 University Medical Center Groningen, Department of Health Sciences, Community and Occupational Medicine, University of Groningen, Groningen, Netherlands

**Keywords:** occupational lung disease, COPD epidemiology

## Abstract

**Objectives:**

To date, only a few studies have investigated the associations between occupational exposures and respiratory outcomes longitudinally in the general population. We investigated the associations between occupational exposures and the development of respiratory symptoms and airway obstruction in the Lifelines Cohort Study.

**Methods:**

We included 35 739 occupationally active subjects with data on chronic cough, chronic phlegm, chronic bronchitis or airway obstruction at baseline and approximately 4.5 years follow-up. Exposures to biological dust, mineral dust, gases/fumes, pesticides, solvents and metals in the current job at baseline were estimated with the ALOHA+job-exposure matrix (JEM). Airway obstruction was defined as FEV_1_/FVC below the lower limit of normal. Logistic regression analysis adjusted for baseline covariates was used to investigate the associations.

**Results:**

At follow-up, 1888 (6.0%), 1495 (4.7%), 710 (2.5%) and 508 (4.5%) subjects had developed chronic cough, chronic phlegm, chronic bronchitis and airway obstruction, respectively. High exposure to biological dust was associated with a higher odds to develop chronic cough and chronic bronchitis. High exposure to pesticides was associated with a higher odds for the development of all respiratory symptoms and airway obstruction. In the multiple exposures analyses, only the association between pesticides exposure and respiratory symptoms remained.

**Conclusions:**

Subjects exposed to high pesticides had a higher odds to develop respiratory symptoms on average 4.5 years later. Control measures should be taken to reduce pesticides exposure among the working population to prevent respiratory symptoms and airway obstruction.

**Key messages**

**What is the key question?**

- Are occupational exposures associated with the development of respiratory symptoms and airway obstruction in the general working population?

**What is the bottom line?**

- High occupational exposure to pesticides is associated with a higher odds to develop respiratory symptoms and airway obstruction in the general working population.

**Why read on?**

- We conducted this study in a large general working population who were followed for a median of 4.5 years and the occupational exposures were estimated with a job-exposure matrix.

## Introduction

In the general population, the prevalence of respiratory symptoms for example, chronic bronchitis (presence of both chronic cough and phlegm) was estimated to be 0%–11%.^1^ Previous studies have reported that chronic bronchitis was associated with an accelerated lung function decline and a higher mortality rate.^2–4^ In 2017, the global prevalence of COPD was estimated to be 3.9%, and the disease accounts for 41.9 deaths per 100 000 subjects which is 5.7% of total all-cause deaths.^5^ Set aside smoking, other factors such as occupational exposures may also impair lung function by stimulating inflammatory responses on inhalation.^6^ Indeed, occupational exposures are responsible for 15%–20% of all COPD cases,^7^ with up to 31% in never smokers.^8^ Therefore, it is important to examine which occupational exposures are associated with the risk to develop respiratory symptoms and airway obstruction in the general population.

A Norwegian study showed an association between exposure to quartz, asbestos and dust/fumes and the development of respiratory symptoms among subjects aged 15–70 years after 11 years of follow-up.^9^ After a follow-up of 20 years, the European Community Respiratory Health Survey (ECRHS) reported that exposure to mineral dust, gases/fumes and metals was associated with a higher risk to develop respiratory symptoms among subjects aged 20–44 years.^10^ Another study with the same population and after the same period of follow-up reported that occupational exposure to biological dust, gases/fumes and pesticides was associated with a 1.5–2.2-fold higher risk to develop airway obstruction.^11^ Consistently, after approximately 11 years of follow-up, a Swiss Cohort Study on Air Pollution and Lung and Heart Diseases in Adults reported that high exposure to biological dust, mineral dust, gases/fumes and vapours, gases, dusts or fumes was associated with a 1.5–4.5-fold higher risk to develop airway obstruction among subjects aged 18–62 years.^12^ Contrary, a recently published Danish nationwide register-based follow-up study showed an inverse association between exposure to biological dust and the development of airway obstruction among subjects aged 19–63 years.^13^ The authors indicated that the lack of smoking data and a healthy worker survivor effect might have biased their results.

In the present study, we investigated the association between airborne exposure to biological dust, mineral dust, gases/fumes, pesticides, solvents and metals (estimated with a job-exposure matrix (JEM)) and the development of chronic cough, chronic phlegm, chronic bronchitis and airway obstruction in >35 000 subjects from the Lifelines Cohort Study who were followed-up for 4.5 years. The Lifelines Cohort Study is a general population-based study investigating subjects from the Northern part of the Netherlands. The strength of the Lifelines Cohort Study lies in the fact that its population is very homogeneous with respect to environmental exposures (eg, air pollution). Additionally, the consistency in regional and cultural work habits will reduce the variability in occupational exposures between people in the same job, compared with studies in which subjects from multiple countries were investigated (eg, the ECRHS).

## Methods

### Population

**Figure 1:**
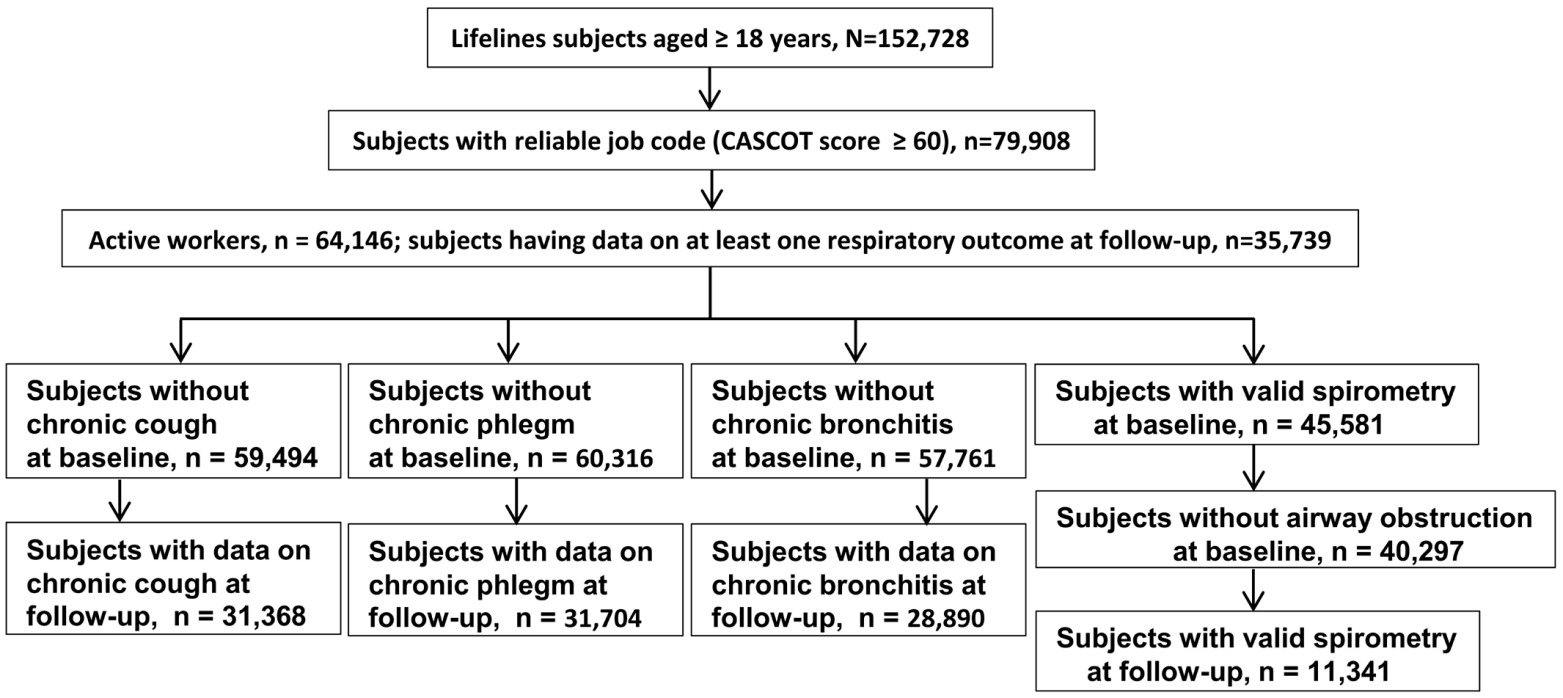
Flowchart of the selection of study subjects. CASCOT, Computer Assisted Structured Coding Tool.

In this study, we included ‘occupationally active workers’ from the Lifelines Cohort Study (figure 1). Baseline data were collected from 2006 to 2013 and the first follow-up visit was conducted between 2014 and 2017 after a median of 4.5 years (range: 1.8–8.8 years). The scientific rationale, study design and survey methods of the Lifelines Cohort Study have been described elsewhere.^14^

### Occupational exposures

We investigated the following occupational exposures: biological dust, mineral dust, gases/fumes, pesticides, solvents and metals. Occupational exposures were estimated using self-reported current job from the baseline questionnaire. The jobs were coded according to the International Standard Classification of Occupations^15^ using a Computer Assisted Structured Coding Tool (CASCOT).^16^ During this procedure, a CASCOT score was given to each coded occupation which indicates the probability that the given code is correct (range: 0–100). We selected subjects with a CASCOT score ≥60, and all job titles above this score were reviewed and, if necessary, recoded to achieve accurate job coding. The ALOHA+JEM^17 18^ was used to link occupational exposures (classified as no, low or high exposure) to the baseline jobs. For details, see online supplemental appendix 1.

10.1136/thoraxjnl-2020-216721.supp1 Supplementary data

### Respiratory outcomes

Chronic cough and chronic phlegm were self-reported both at baseline and follow-up, using the ECRHS questionnaire (for definitions, see online supplemental appendix 2).^19^ Chronic bronchitis was defined as the presence of both chronic cough and chronic phlegm. At baseline and follow-up, lung function was measured by prebronchodilator spirometry according to American Thoracic Society/European Respiratory Society (ATS/ERS) guidelines^20^ using the Welch AllynSpiroPerfect device (Welch Allyn V.1.6.0.489, PC-based SpiroPerfect with CardioPerfect Workstation software). Airway obstruction was defined as the ratio of FEV_1_/FVC <lower limit of normal.^21^ Due to practical reasons, spirometry was performed in a random subset of the Lifelines participants.

### Covariates

The subjects’ age and sex were taken from the baseline screening. Education, monthly income, smoking status and pack-years were extracted from the baseline questionnaires.

### Statistical analyses

Population characteristics were analysed for occupationally active subjects at baseline with data on at least one respiratory outcome at follow-up. In the current study, we used a follow-up design in which the exposure precedes the outcome, and thus only information about occupational exposures and covariates at baseline was included. To assess the correlation between occupational exposures, a non-parametric Spearman’s rank-order correlation was used. To investigate the risk to develop chronic cough, we excluded subjects who reported chronic cough at baseline. Similarly, to investigate the risk to develop chronic sputum, chronic bronchitis and airway obstruction, we excluded subjects with chronic sputum, chronic bronchitis and airway obstruction, respectively, at baseline. Logistic regression was used to investigate the association between occupational exposures (no exposure to the specific agent as reference) and respiratory outcomes (chronic cough, chronic phlegm, chronic bronchitis and airway obstruction) at follow-up, adjusting for age, sex, education, monthly income, pack-years and smoking status (the type and categories of each covariate are given in table 1). All exposures were initially tested separately. Subsequently, we entered all exposures in one model to adjust for coexposures. A two-sided p value <0.05 was considered statistically significant.

**Table 1:** Population characteristics of symptom-free subjects at baseline

Population characteristics, n=35 739	
Age (years), mean (SD)	43 (10)
Females (%)	59.8
Education	
Low, n (%)	4365 (12.3)
Medium, n (%)	18 467 (52.2)
High, n (%)	12 016 (34.0)
Unclassifiable, n (%)	504 (1.5)
Monthly income	
Low, n (%)	4231 (12.0)
Medium, n (%)	9416 (26.6)
High, n (%)	17 189 (48.8)
Not reported, n (%)	4364 (12.6)
Pack-years in ever smokers, median (IQR)	8 (12)
Smoking	
Never smoker, n (%)	16 979 (48.8)
Ex-smoker, n (%)	11 259 (32.4)
Current smoker, n (%)	6541 (18.8)
FEV_1_% predicted, mean (SD)	96.0 (12.6)
FVC% predicted, mean (SD)	100.1 (12.0)
FEV_1_/FVC% predicted, mean (SD)	95.4 (7.8)
Biological dust	
No, n (%)	23 252 (65.7)
Low, n (%)	10 353 (29.3)
High, n (%)	1774 (5.0)
Mineral dust	
No, n (%)	28 094 (79.4)
Low, n (%)	5279 (14.9)
High, n (%)	2006 (5.7)
Gases/fumes	
No, n (%)	19 228 (54.3)
Low, n (%)	13 993 (39.6)
High, n (%)	2158 (6.1)
Pesticides	
No, n (%)	33 553 (94.8)
Low, n (%)	1398 (4.0)
High, n (%)	428 (1.2)
Solvents	
No, n (%)	24 956 (70.5)
Low, n (%)	8858 (25.0)
High, n (%)	1565 (4.5)
Metals	
No, n (%)	33 643 (95.1)
Low, n (%)	1004 (2.8)
High, n (%)	732 (2.1)

**Education**: low education (no training, primary education, lower or prevocational education); medium education (general secondary education, secondary vocational or professional guiding, preuniversity education); high education (higher professional or university degree); unclassifiable (subjects with other than above-mentioned education).

**Monthly income**: low-income (monthly net income ≤ €1500); medium-income (monthly net income between €1500 up and €2500); high-income (monthly net income ≥ €2500); unknown (I do not know/I do not want to say).

**Smoking**: never smokers (never smoked or smoked for <1 year); ex-smokers (smoked for ≥1 year and stopped smoking for ≥1 month); current smokers (current smoker or stopped smoking <1 month).

FEV_1_, forced expiratory volume in 1 second; FVC, forced vital capacity.

### Sensitivity analyses

To assess if the associations between occupational exposure and symptom development remain consistent if we use a more strict inclusion of asymptomatic subjects at baseline, we performed sensitivity analyses. We investigated the association between airborne occupational exposures and the development of respiratory symptoms including only subjects without both chronic cough and chronic phlegm at baseline. We additionally investigated each exposure in comparison to a common control group consisting of subjects with no exposure to any of the six occupational exposures under study. Finally, we investigated the association between occupational exposures and the development of respiratory symptoms and airway obstruction in subjects without asthma at baseline.

## Results

### Baseline characteristics

Table 1 shows the baseline characteristics of the subjects having data on at least one respiratory outcome (n=35 739). At baseline, the mean age of the population was 43 years (SD: 10 years) and the majority was women (59.8%). Approximately half of the subjects had received a medium education and a high monthly income. The median pack-years in ever smokers was 8 (IQR: 12) and about half of the subjects were ever smoker.

Exposure to gases/fumes was most prevalent (45.7% with low or high exposure) followed by exposure to biological dust (34.3%), and exposure to solvents (29.5%) (table 1). Exposure to metals (4.9%) and exposure to pesticides (5.2%) were least prevalent. The prevalence of occupational exposures stratified by the respiratory outcomes is given in online supplemental table E1.

**Figure 2:**
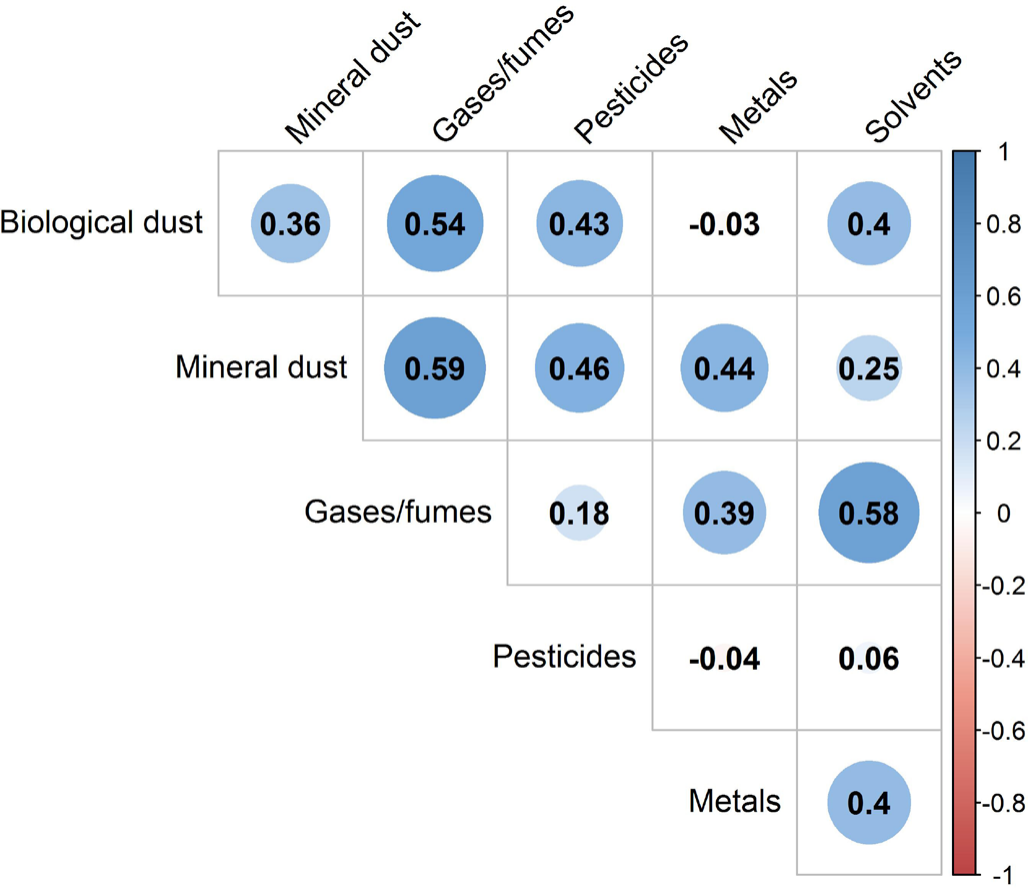
Correlogram shows the correlation among occupational exposures at baseline.

The Spearman rank correlation between baseline occupational exposures is shown in figure 2. The highest correlations were found between exposure to gases/fumes and biological dust (r=0.54), mineral dust (r=0.59) and solvents (r=0.58). The weakest correlations were seen between exposure to pesticides and metals and solvents and between biological dust and metals.

### Occupational exposures and the development of respiratory symptoms and airway obstruction

At follow-up, 1888 (6.0%) subjects had developed chronic cough, 1495 (4.7%) subjects had developed chronic phlegm, 710 (2.5%) subjects had developed chronic bronchitis and 508 (4.5%) subjects had developed airway obstruction.

In the adjusted models, high exposure to pesticides was associated with a 1.5–2.2-fold higher odds to develop respiratory symptoms and airway obstruction (table 2). In addition, high exposure to biological dust was associated with a significant 1.3–1.5-fold higher odds to develop chronic cough and chronic bronchitis. In the model with all six airborne exposures, the odds estimates for high exposure to pesticides increased and remained statistically significant for chronic phlegm and chronic bronchitis (table 3).

**Table 2:** Associations between occupational exposures and the development of respiratory symptoms and airway obstruction

Occupational exposures	N (31 368)	Chronic cough	N (31 704)	Chronic phlegm	N (28 890)	Chronic bronchitis	N (11 341)	Airway obstruction
		OR (95% CI)		OR (95% CI)		OR (95% CI)		OR (95% CI)
Biological dust								
No	20 630	Ref.	20 845	Ref.	18 987	Ref.	7572	Ref.
Low	9191	0.95 (0.85 to 1.06)	9317	0.93 (0.82 to 1.05)	8524	0.96 (0.80 to 1.15)	3226	1.17 (0.95 to 1.43)
High	1547	**1.26 (1.03 to 1.54)**	1542	1.08 (0.86 to 1.36)	1379	**1.46 (1.07 to 1.99)**	543	1.17 (0.75 to 1.81)
Mineral dust								
No	25 030	Ref.	25 310	Ref.	23 181	Ref.	9099	Ref.
Low	4605	0.92 (0.80 to 1.07)	4664	1.01 (0.87 to 1.18)	4193	1.02 (0.82 to 1.27)	1640	0.74 (0.54 to 1.00)
High	1733	1.21 (1.00 to 1.46)	1730	1.08 (0.87 to 1.35)	1516	1.08 (0.78 to 1.49)	602	1.20 (0.80 to 1.79)
Gases/fumes								
No	17 188	Ref.	17 388	Ref.	15 927	Ref.	6274	Ref.
Low	12 322	1.05 (0.94 to 1.17)	12 452	0.99 (0.87 to 1.11)	11 320	1.11 (0.93 to 1.32)	4413	1.09 (0.89 to 1.33)
High	1858	1.15 (0.94 to 1.40)	1864	1.04 (0.83 to 1.30)	1643	1.16 (0.84 to 1.59)	654	1.15 (0.76 to 1.74)
Pesticides								
No	29 767		30 095	Ref.	27 446	Ref.	10 815	Ref.
Low	1227	1.19 (0.95 to 1.50)	1232	1.03 (0.79 to 1.34)	1110	1.36 (0.95 to 1.93)	406	1.14 (0.69 to 1.87)
High	374	**1.45 (1.01 to 2.07)**	377	**1.49 (1.01 to 2.20)**	334	**1.99 (1.19 to 3.31)**	120	**2.24 (1.14 to 4.39)**
Solvents								
No	22 119	Ref.	22 390	Ref.	20 387	Ref.	8019	Ref.
Low	7892	1.10 (0.98 to 1.23)	7961	1.01 (0.89 to 1.14)	7292	1.12 (0.93 to 1.33)	2828	1.16 (0.94 to 1.43)
High	1357	1.08 (0.86 to 1.34)	1353	1.06 (0.83 to 1.36)	1211	0.92 (0.64 to 1.34)	494	1.03 (0.66 to 1.62)
Metals								
No	29 877	Ref.	30 216	Ref.	27 574	Ref.	10 784	Ref.
Low	868	1.06 (0.81 to 1.37)	868	1.11 (0.83 to 1.48)	775	1.21 (0.81 to 1.80)	338	0.72 (0.39 to 1.34)
High	623	1.10 (0.82 to 1.48)	620	1.00 (0.71 to 1.41)	541	0.73 (0.41 to 1.29)	219	0.99 (0.51 to 1.92)

Bold values are p<0.05.

The logistic regression was adjusted for baseline age, sex, education, monthly income, pack-years and smoking. No exposureas reference group.

**Table 3:** Associations between occupational exposures and the development of respiratory symptoms and airway obstruction; coexposures analyses

Occupational exposures	N (31 368)	Chronic cough	N (31 704)	Chronic phlegm	N (28 890)	Chronic bronchitis	N (11 341)	Airway obstruction
		OR (95% CI)		OR (95% CI)		OR (95% CI)		OR (95% CI)
Biological dust								
No	20 630	Ref.	20 845	Ref.	18 987	Ref.	7572	Ref.
Low	9191	0.86 (0.74 to 1.01)	9317	0.88 (0.74 to 1.05)	8524	0.83 (0.64 to 1.07)	3226	1.20 (0.87 to 1.64)
High	1547	1.10 (0.82 to 1.47)	1542	1.08 (0.77 to 1.51)	1379	1.18 (0.75 to 1.86)	543	0.99 (0.53 to 1.86)
Mineral dust								
No	25 030	Ref.	25 310	Ref.	23 181	Ref.	9099	Ref.
Low	4605	0.88 (0.73 to 1.05)	4664	1.03 (0.84 to 1.25)	4193	0.90 (0.68 to 1.19)	1640	0.81 (0.41 to 1.1.33)
High	1733	1.01 (0.77 to 1.33)	1730	0.96 (0.71 to 1.32)	1516	0.73 (0.46 to 1.15)	602	0.77 (0.42 to 1.42)
Gases/fumes								
No	17 188	Ref.	17 388	Ref.	15 927	Ref.	6274	Ref.
Low	12 322	1.06 (0.90 to 1.25)	12 452	1.01 (0.84 to 1.21)	11 320	1.16 (0.89 to 1.51)	4413	1.08 (0.79 to 1.47)
High	1858	1.22 (0.93 to 1.60)	1864	1.05 (0.77 to 1.42)	1643	1.45 (0.95 to 2.23)	654	1.58 (0.90 to 2.79)
Pesticides								
No	29 767	Ref.	30 095	Ref.	27 446	Ref.	10 815	Ref.
Low	1227	1.15 (0.82 to 1.60)	1232	0.98 (0.67 to 1.44)	1110	1.28 (0.76 to 2.15)	406	1.41 (0.69 to 2.87)
High	374	1.37 (0.88 to 2.13)	377	**1.64 (1.01 to 2.67)**	334	**2.58 (1.32 to 5.07)**	120	2.31 (0.94 to 5.70)
Solvents								
No	22 119	Ref.	22 390	Ref.	20 387	Ref.	8019	Ref.
Low	7892	1.12 (0.95 to 1.32)	7961	1.03 (0.86 to 1.23)	7292	1.06 (0.82 to 1.38)	2828	0.99 (0.71 to 1.38)
High	1357	1.07 (0.81 to 1.41)	1353	1.09 (0.80 to 1.48)	1211	0.92 (0.59 to 1.45)	494	1.00 (0.58 to 1.73)
Metals								
No	29 877	Ref.	30 216	Ref.	27 574	Ref.	10 784	Ref.
Low	868	1.01 (0.74 to 1.36)	868	1.10 (0.79 to 1.53)	775	1.25 (0.78 to 1.98)	338	0.77 (0.38 to 1.54)
High	623	0.98 (0.66 to 1.47)	620	0.96 (0.61 to 1.53)	541	0.81 (0.40 to 1.66)	219	0.90 (0.37 to 2.18)

Bold values are p<0.05.

The logistic regression was adjusted for baseline age, sex, education, monthly income, pack-years, smoking and coexposures. No exposure as reference group.

### Sensitivity analyses

The associations between occupational exposures and the development of respiratory symptoms among subjects without both chronic cough and without chronic phlegm at baseline were comparable to the main findings (online supplemental table E2). In addition, the associations between occupational exposures and the development of respiratory symptoms and airway obstruction in comparison to a common control group consisting of subjects with no exposure to any of the six occupational agents were not notably different from the main findings (online supplemental table E3). The associations between occupational exposures and the development of respiratory symptoms and airway obstruction in subjects without asthma at the baseline were comparable to the main findings (online supplemental table E4).

## Discussion

### Main findings

After a median follow-up of 4.5 years in the Lifelines Cohort Study, we found that subjects were at a higher odds to develop respiratory outcomes (chronic cough, chronic phlegm, chronic bronchitis and airway obstruction) on high occupational exposure to biological dust and pesticides. Mutual adjustment for the other exposures showed that only high pesticide exposure was persistently associated with the outcomes.

### Occupational exposures and the development of respiratory symptoms and airway obstruction

We found that in the single exposure model, high occupational exposure to pesticides was associated with a higher odds to develop airway obstruction at follow-up. In line with our current findings, the ECRHS study found that in the single exposure model, exposure to pesticides was associated with a higher risk to develop airway obstruction after a follow-up of 20 years.^11^ A previous cross-sectional study within Lifelines also found that pesticides exposure was associated with a higher prevalence of airway obstruction.^22^ Our current findings strengthen the evidence that occupational exposure to pesticides is associated with a higher risk to develop airway obstruction in the general working population. Interestingly, in the coexposure model, the odds of the association between exposure to pesticides and airway obstruction increased (single exposure model vs coexposure model: 2.12 vs 2.23), but became borderline (p=0.091) significant.

Further, we found that high pesticides exposure is also a risk factor for developing respiratory symptoms. In contrast, the ECRHS study found no association between exposure to pesticides and the development of respiratory symptoms in the general population after 20 years of follow-up.^10^ Compared with the ECRHS study, in the current study, we included subjects with a wider age range (18–65 years vs 20–44 years), but the follow-up period is much shorter than in the ECRHS study (4.5 years vs 20 years). In addition, we did not incorporate cumulative exposure in our exposure estimate as was done in the ECRHS study. Furthermore, large heterogeneity may exist in exposure to pesticides across the 30 centres of 15 European countries in the ECRHS study, whereas in our study all participants came from the three northern provinces of the Netherlands with considerably more farmers than in the more urbanised parts of the Netherlands. The participating ECRHS centres were also mainly urban centres and consequently, the number of farmers within the ECRHS study is relatively low. These discrepancies might explain the difference in the effect of pesticides exposure on respiratory health between the current study and the ECRHS study.

In the ECRHS study, metals exposure was associated with a higher risk to develop respiratory symptoms which was not the case in our study. The prevalence of metals exposure was higher in the ECRHS than in Lifelines (≈11% vs ≈5%). There is not much heavy industry in the northern provinces of the Netherlands which could explain the low prevalence of metals exposure. In addition, in the Lifelines Cohort Study, women are over-represented (≈60%), and not many females work in the metals industry.

The odds of developing respiratory symptoms and airway obstruction after being exposed to high pesticides remained significant in the analyses with adjustment for multiple exposures and even became somewhat stronger. This indicates that high exposure to pesticides at the workplace is the main driver of developing respiratory symptoms and airway obstruction among workers within Lifelines. Pesticides cover various chemical substances. To date, the biological mechanism through which the different active ingredients in pesticides affect the airways is poorly understood. A previous study reported that certain pesticides may induce inflammation by increasing neutrophil reactive oxygen molecule production and interleukin-8 secretion.^23^ In addition, organophosphates and carbamates inhibit acetylcholinesterase, which results in mucus hypersecretion and airway smooth muscle contraction.^24^ Thus, persistent inflammation induced by pesticides might result in chronic respiratory symptoms and airway obstruction.

Previously, we found that occupational exposure to pesticides was associated with a lower level of inflammatory biomarkers (C reactive protein and neutrophils), and was not associated with changes in biomarkers after 4.5 years follow-up.^25^ This finding indicates that pesticides are not leading to higher levels of inflammation and may thus alter or damage our immune system through other biological pathways. Cytokines pathways, induction of oxidative stress, mitochondrial dysfunction, endoplasmic reticulum stress, disruption of the ubiquitin protease system or autophagy, inhibition of enzymes with esterase activity, and endocrine disruption are some suggested biological pathways through which pesticides could disrupt the immune system.^26 27^ Further research is required to shed light on the respiratory health risks of specific active ingredients of pesticides, the biological mechanism, and the exposure–response relationship.

In the analyses without adjustment for coexposure, we found that symptom-free individuals with high biological dust exposure had a higher odds of developing chronic cough and chronic bronchitis. These significant associations between high biological dust exposure and the development of symptoms disappeared in the analyses with all exposures and only the association between high pesticides exposure and symptom development remained. We observed that all pesticide exposed workers, for example, crop growers (n≈110), gardeners (n≈110), animal producers (n≈530) and labours in agriculture and forest (n≈5), were also exposed to biological dust, but not all biological dust exposed workers were exposed to pesticides (eg, fibre preparers, weavers, knitters and paper-making plants operators). We examined the association between exposure to biological dust and the development of respiratory symptoms among these subjects who were exposed only to biological dust but not to pesticides. The analyses showed no significant association between biological dust exposure and the development of respiratory symptoms (online supplemental table E5). This indicates that the significant association between exposure to biological dust and respiratory symptoms was confounded by exposure to pesticides.

In the general population-based Vlagtwedde-Vlaardingen study, we showed that pesticides exposure was associated with accelerated lung function decline after 25 years of follow-up in the 70s.^28^ In 1979, the guidelines on pesticides were first legislated at the European Union level, and have evolved considerably over the years.^29^ Thus, our current study findings indicate that the existing policies and legislation on pesticides may still not be adequate to protect the workers from the adverse respiratory health effects of occupational exposure to pesticides. Recent studies conducted in low-income and middle-income countries also showed that farmers with pesticides exposure were at a higher risk of developing respiratory symptoms and airway obstruction.^30–32^

### Strengths and limitations

In this study, we included a very large number of occupationally active subjects covering a wide age range and followed for a median of 4.5 years from the Lifelines Cohort Study. Lifelines contains a large amount of quality data which allowed us to adjust for important confounders. Our study population is homogenous in terms of ethnicity, geographical locations and other environmental exposures such as air pollution, and therefore, our study findings are not confounded by these factors. The participants of the Lifelines Cohort Study are representative of the general population of the three northern provinces of the Netherlands.^33^ In addition, data on respiratory symptoms were measured with a validated questionnaire and lung function was measured according to a standardised protocol. We performed additional analyses to assess the effect of coexposure in our findings.

We used the expert-based ALOHA+JEM to estimate occupational exposure. We prefer the use of a JEM over self-reported exposure since workers often struggle to recall detailed information on working conditions many years back, and in many instances, they link their disease condition with previous exposure (recall bias). An objectively constructed JEM is a more robust tool in estimating occupational exposure and eliminating differential bias.^34^ A JEM by definition does not account for differences in exposure levels observed between individuals with the same reported job.^35^ However, since our study population is from the same geographical region, the regional and cultural work habits will minimise this variability in occupational exposures between individuals with the same job. The ALOHA+JEM also does not assess exposure at the individual chemical or biological level. All these shortcomings may lead to imprecision, but due to the Berkson nature of this error, the presented odds estimates will be hardly or not biased.^36^ In this study, we used the information about occupational exposure at baseline and did not take into account potential changes in occupational exposures between baseline and follow-up. Given that the duration of follow-up is relatively short (median 4.5 years), we do not expect that many people have changed their jobs, however, we cannot entirely rule out the possible impact of these changes on the outcomes. In addition, the findings of this study are based on a homogenous population from the northern Netherlands, which may limit the generalisability to other populations to some extent.

## Conclusion

In this study, we found that high occupational exposure to pesticides was associated with a higher odds of developing respiratory symptoms and airway obstruction among the general working population. More rigorous efforts are required to protect workers from the adverse health effects of occupational pesticides exposure. This can be done by adopting a hierarchy of pesticide control measures, starting from a reduction or elimination of pesticides in the workplace to substitution by alternative materials and to the engineering of control measures (eg, enclosure or isolation of the hazardous work process) and administrative measures (eg, routine surveillance of the safety management system and guidelines).

## Data Availability

Data may be obtained from a third party and are not publicly available. Registration is required to obtain data from the Lifelines Cohort Study. It is not permitted to deposit the Lifelines data in an open data repository. To obtain data, used in the current study, interested researchers should contact the Lifelines Cohort Study (www.lifelines.nl).
