# Supplementary material for: Airborne occupational exposures and the risk of developing respiratory symptoms and airway obstruction in the Lifelines Cohort Study
Source: Thorax. 2021 Mar 2;76(8):790–7. doi: 10.1136/thoraxjnl-2020-216721 (PMC8311115; doi:10.1136/thoraxjnl-2020-216721)
Supplement: Supplementary data [file thoraxjnl-2020-216721supp001.pdf]

# Airborne occupational exposures and the risk of developing respiratory symptoms and airway obstruction in the Lifelines Cohort study

Md Omar Faruque, H Marike Boezen, Hans Kromhout, Roel Vermeulen, Ute Bültmann, Judith M Vonk

## Online supplementary material

**Appendix 1: Conversion of ISCO-08 into ISCO-88.**

To be able to use the ALOHA+ JEM to estimate the level of occupational exposure, we converted the ISCO-08 codes into ISCO-88 codes. For this conversion, we used the conversion table provided by the International Labour Organization and the conversion SPSS-syntax provided by Ganzeboom and Treiman. For ISCO-08 codes that could be converted into multiple ISCO-88 codes, we selected the ISCO-88 code with the highest exposure estimate. For example, ISCO-08 code 5164 (Pet groomers and animal care workers) could be recoded to either ISCO-88 5139 (Personal care and related workers not elsewhere classified) or ISCO-88 6129 (Market-oriented animal producers and related workers not elsewhere classified). As 6129 had higher levels of estimated exposures according to the JEM than 5139, we recoded ISCO-08 5164 into ISCO-88 6129. In addition, some categories were combined in ISCO-08 compared to ISCO-88 (e.g., ISCO-08 8160 - Food and related product machine operators - had different codes in ISCO-88 depending on the type of food/product - ISCO-88 8271 till 8279). For these combined categories, we reviewed the types of jobs and manually recoded them to the correct ISCO-88 category. After this conversion, the ALOHA+ JEM was used to estimate the occupational exposure into no, low, or high exposure categories (0/1/2) for all occupational exposures: biological dust, mineral dust, gases and fumes, insecticides, herbicides, fungicides, aromatic solvents, chlorinated solvents, other solvents, and heavy metals.



Faruque MO, *et al. Thorax* 2021; 76:790–797. doi: 10.1136/thoraxjnl-2020-216721

**Appendix 2: Definition of respiratory symptoms.**

Chronic cough was defined as “Usual coughing first thing in the morning, or during the day, or at night in winter for at least three months in a year.”

Chronic phlegm was defined as “Usual bringing up any phlegm first thing in the morning, or during the day, or at night in winter for at least three months in a year.



Faruque MO, *et al. Thorax* 2021; 76:790–797. doi: 10.1136/thoraxjnl-2020-216721

**Supplementary table E1: Distribution of the occupational exposures at baseline stratified by respiratory outcomes.**

| Occupational exposures | Chronic cough (n=31,368) | | | | | | Chronic phlegm (31,704) | | | | | |
|---|---|---|---|---|---|---|---|---|---|---|---|---|
| | No (n=29,480) | | | Yes (n=1,888) | | | No (n=30,209) | | | Yes (n=1,495) | | |
| | No | Low | High | No | Low | High | No | Low | High | No | Low | High |
| | n % | n % | n % | n % | n % | n % | n % | n % | n % | n % | n % | n % |
| **Biological dust** | 19410 (65.8) | 8660 (29.4) | 1410 (4.8) | 1220 (64.6) | 531 (28.1) | 137 (7.3) | 19859 (65.7) | 8904 (29.5) | 1446 (4.8) | 986 (66.0) | 413 (27.6) | 96 (6.4) |
| **Mineral dust** | 23620 (80.1) | 4289 (14.5) | 1571 (5.3) | 1410 (74.7) | 316 (16.7) | 162 (8.6) | 24197 (80.1) | 4400 (14.6) | 1612 (5.3) | 1113 (74.4) | 264 (17.7) | 118 (7.9) |
| **Gases/fumes** | 16259 (55.2) | 11528 (39.1) | 1693 (5.7) | 929 (49.2) | 794 (42.1) | 165 (8.7) | 166636 (55.1) | 11833 (39.2) | 1740 (5.8) | 752 (50.3) | 619 (41.4) | 124 (8.3) |
| **Pesticides** | 28014 (95.0) | 1130 (3.8) | 336 (1.1) | 1753 (92.8) | 97 (5.1) | 38 (2.0) | 28699 (95.0) | 1165 (3.9) | 345 (1.1) | 1396 (93.4) | 67 (4.5) | 32 (2.1) |
| **Solvents** | 20829 (70.7) | 7393 (25.1) | 1258 (4.3) | 1290 (68.3) | 499 (26.4) | 99 (5.2) | 21347 (70.7) | 7587 (25.1) | 1275 (4.2) | 1043 (69.8) | 374 (25.0) | 78 (5.2) |
| **Metals** | 28114 (95.4) | 797 (2.7) | 569 (1.9) | 1763 (93.8) | 71 (3.8) | 54 (2.9) | 28821 (95.4) | 807 (2.7) | 581 (1.9) | 1395 (93.3) | 61 (4.1) | 39 (2.6) |

| CONTINUED | | | | | | | | | | | | |
|---|---|---|---|---|---|---|---|---|---|---|---|---|
| Occupational exposures | Chronic bronchitis (28,890) | | | | | | Airway obstruction (n=11,341) | | | | | |
| | No (28180) | | | Yes (710) | | | No (n=10830) | | | Yes (n=508) | | |
| | No | Low | High | No | Low | High | No | Low | High | No | Low | High |
| | n % | n % | n % | n % | n % | n % | n % | n % | n % | n % | n % | n % |
| **Biological dust** | 18537 (65.8) | 8319 (29.5) | 1324 (4.7) | 450 (63.4) | 205 (28.9) | 55 (7.7) | 7248 (66.9) | 3065 (28.3) | 517 (4.8) | 322 (63.4) | 160 (31.5) | 26 (5.1) |
| **Mineral dust** | 22657 (80.4) | 4062 (14.4) | 1461 (5.2) | 524 (73.8) | 131 ( 18.5) | 55 (7.7) | 8686 (80.2) | 1577 (14.6) | 567 (5.2) | 410 (80.7) | 63 (12.4) | 35 (6.9) |
| **Gases/fumes** | 15592 (55.3) | 11004 (39.0) | 1584 (5.6) | 335 (47.2) | 316 (44.5) | 59 (8.3) | 6015 (55.5) | 4195 (38.7) | 620 (5.7) | 257 (50.6) | 217 (42.7) | 34 (6.7) |
| **Pesticides** | 26793 (95.1) | 1072 (3.8) | 315 (1.1) | 653 (92.0) | 38 (5.4) | 19 (2.7) | 10333 (95.8) | 387 (3.6) | 110 (1.0) | 479 (94.3) | 19 (3.7) | 10 (2.0) |
| **Solvents** | 19905 (70.6) | 7097 (25.2) | 1178 (4.2) | 482 (67.9) | 195 (27.5) | 33 (4.6) | 7675 (70.9) | 2686 (24.8) | 469 (4.3) | 342 (67.3) | 141 (27.8) | 25 (4.9) |
| **Metals** | 26909 (95.5) | 744 (2.6) | 527 (1.9) | 665 (93.7) | 31 (4.4) | 14 (2.0) | 10295 (95.1) | 327 (3.0) | 208 (1.9) | 486 (95.7) | 11 (2.2) | 11 (2.2) |



Faruque MO, *et al. Thorax* 2021; 76:790–797. doi: 10.1136/thoraxjnl-2020-216721

**Supplementary table E2: Association between occupational exposures and the development of respiratory symptoms in subjects without any respiratory symptoms at baseline. The logistic regression was adjusted for baseline age, sex, education, monthly income, pack-years, and smoking.**

| Occupational exposures | N (30491) | Chronic cough OR (95%CI) | N (30490) | Chronic phlegm OR (95%CI) | N (28890) | Chronic bronchitis OR (95%CI) |
|---|---|---|---|---|---|---|
| **Biological dust** | | | | | | |
| No | 20055 | Ref. | 20052 | Ref. | 18987 | Ref. |
| Low | 8957 | 0.94 (0.84;1,06) | 8958 | 0.91 (0.79;1.04) | 8524 | 0.96 (0.80;1.15) |
| High | 1479 | 1.22 (0.99;1.51) | 1480 | 1.16 (0.91;1.49) | 1379 | **1.46 (1.07;1.99)** |
| **Mineral dust** | | | | | | |
| No | 24395 | Ref. | 24394 | Ref. | 23181 | Ref. |
| Low | 4442 | 0.92 (0.79;1.07) | 4445 | 0.98 (0.83;1.16) | 4193 | 1.02 (0.82;1.27) |
| High | 1654 | 1.18 (0.96;1.44) | 1651 | 1.07 (0.85;1.36) | 1516 | 1.08 (0.78;1.49) |
| **Gases and fumes** | | | | | | |
| No | 16763 | Ref. | 16761 | Ref. | 15927 | Ref. |
| Low | 11952 | 1.04 (0.93;1.16) | 11952 | 0.98 (0.86;1.12) | 11320 | 1.11 (0.93;1.32) |
| High | 1776 | 1.17 (0.95;1.43) | 1777 | 0.97 (0.76;1.24) | 1643 | 1.16 (0.84;1.59) |
| **Pesticides** | | | | | | |
| No | 28954 | Ref. | 28952 | Ref. | 27446 | Ref. |
| Low | 1174 | 1.09 (0.86;1.41) | 1176 | 1.07 (0.81;1.43) | 1110 | 1.36 (0.95;1.93) |
| High | 363 | **1.48 (1.03;2.14)** | 362 | **1.65 (1.11;2.47)** | 334 | **1.99 (1.19;3.31)** |
| **Solvents** | | | | | | |
| No | 21508 | Ref. | 21506 | Ref. | 20387 | Ref. |
| Low | 7680 | 1.08 (0.97;1.22) | 7680 | 0.99 (0.87;1.14) | 7292 | 1.12 (0.94;1.33) |
| High | 1303 | 1.11 (0.88;1.39) | 1304 | 1.01 (0.77;1.32) | 1211 | 0.92 (0.64;1.34) |
| **Metals** | | | | | | |
| No | 29071 | Ref. | 29069 | Ref. | 27574 | Ref. |
| Low | 830 | 1.14 (0.87;1.49) | 831 | 1.05 (0.77;1.43) | 775 | 1.21 (0.81;1.80) |
| High | 590 | 1.08 (0.79;1.48) | 590 | 0.83 (0.56;1.23) | 541 | 0.73 (0.41;1.29) |

OR= Odds ratio; CI=Confidence interval, No exposure as reference group



Faruque MO, *et al. Thorax* 2021; 76:790–797. doi: 10.1136/thoraxjnl-2020-216721

**Supplementary table E3: Association between occupational exposures and the development of respiratory symptoms and airway obstruction. The logistic regression was adjusted for baseline age, sex, education, monthly income, pack-years, and smoking. In this model, each exposure was compared to a common group consisting of no exposure to all six occupational agents under study.**

| Occupational exposures | N (26842) | Chronic cough | N (27147) | Chronic phlegm | N (24815) | Chronic bronchitis | N (9658) | Airway obstruction |
|---|---|---|---|---|---|---|---|---|
| | | OR (95%CI) | | OR (95%CI) | | OR (95%CI) | | OR (95%CI) |
| **Biological dust** | | | | | | | | |
| No | 16104 | Ref. | 16288 | Ref. | 14912 | Ref. | 5889 | Ref. |
| Low | 9191 | 1.00 (0.88;1.13) | 9317 | 0.96 (0.83;1.1) | 8524 | 1.06 (0.87;1.29) | 3226 | 1.15 (0.92;1.44) |
| High | 1547 | **1.34 (1.08;1.66)** | 1542 | 1.15 (0.9;1.47) | 1379 | **1.70 (1.22;2.37)** | 543 | 1.14 (0.72;1.80) |
| **Mineral dust** | | | | | | | | |
| No | 16104 | Ref. | 16288 | Ref. | 14912 | Ref. | 5889 | Ref. |
| Low | 4605 | 0.96 (0.82;1.12) | 4664 | 1.01 (0.85;1.20) | 4193 | 1.10 (0.85;1.41) | 1640 | 0.86 (0.62;1.19) |
| High | 1733 | **1.25 (1.01;1.54)** | 1730 | 1.07 (0.84;1.36) | 1516 | 1.15 (0.81;1.63) | 602 | 1.42 (0.92;2.19) |
| **Gases and fumes** | | | | | | | | |
| No | 16104 | Ref. | 16288 | Ref. | 14912 | Ref. | 5889 | Ref. |
| Low | 12322 | 1.05 (0.94;1.18) | 12452 | 0.99 (0.88;1.13) | 11320 | 1.13 (0.95;1.36) | 4413 | 1.10 (0.89;1.35) |
| High | 1858 | 1.15 (0.94;1.40) | 1730 | 1.03 (0.82;1.29) | 1643 | 1.17 (0.85;1.61) | 654 | 1.16 (0.76;1.76) |
| **Pesticides** | | | | | | | | |
| No | 16104 | Ref. | 16288 | Ref. | 14912 | Ref. | 5889 | Ref. |
| Low | 1227 | 1.24 (0.97;1.59) | 1232 | 1.08 (0.81;1.44) | 1110 | 1.60 (1.08;2.36) | 406 | 1.18 (0.70;2.00) |
| High | 374 | **1.52 (1.05;2.20)** | 377 | **1.57 (1.05;2.35)** | 334 | **2.35 (1.37;4.02)** | 120 | **2.28 (1.13;4.59)** |
| **Solvents** | | | | | | | | |
| No | 16104 | Ref. | 16288 | | 14912 | Ref. | 5889 | Ref. |
| Low | 7892 | 1.11 (0.98;1.25) | 7961 | 1.02 (0.89;1.17) | 7292 | 1.20 (0.99;1.46) | 2828 | 1.16 (0.93;1.45) |
| High | 1357 | 1.09 (0.87;1.38) | 1353 | 1.09 (0.84;1.41) | 1211 | 1.05 (0.71;1.55) | 494 | 1.05 (0.66;1.67) |
| **Metals** | | | | | | | | |
| No | 16104 | Ref. | 16288 | Ref. | 14912 | Ref. | 5889 | Ref. |
| Low | 868 | 1.10 (0.83;1.45) | 868 | 1.16 (0.85;1.58) | 775 | 1.38 (0.89;2.14) | 338 | 0.81 (0.43;1.55) |
| High | 623 | 1.14 (0.83;1.58) | 620 | 1.06 (0.73;1.53) | 541 | 0.85 (0.46;1.57) | 219 | 1.18 (0.58;2.43) |

OR=Odds ratio; CI=Confidence interval, No exposure as reference group



Faruque MO, *et al. Thorax* 2021; 76:790–797. doi: 10.1136/thoraxjnl-2020-216721

**Supplementary table E4: Association between occupational exposures and the development of respiratory symptoms and airway obstruction in subjects without asthma. The logistic regression was adjusted for baseline age, sex, education, monthly income, pack-years, and smoking.**

| Occupational exposures | N (28990) | Chronic cough OR (95%CI) | N (29275) | Chronic phlegm OR (95%CI) | N (26865) | Chronic bronchitis OR (95%CI) | N (10557) | Airway obstruction OR (95%CI) |
|---|---|---|---|---|---|---|---|---|
| **Biological dust** | | | | | | | | |
| No | 19066 | Ref. | 19235 | Ref. | 17644 | Ref. | 7032 | Ref. |
| Low | 8476 | 0.93 (0.82;1.05) | 8589 | 0.95 (0.83;1.09) | 7918 | 1.00 (0.83;1.22) | 3014 | 1.19 (0.96;1.48) |
| High | 1448 | **1.25 (1.01;1.55)** | 1451 | 1.17 (0.92;1.50) | 1303 | **1.55 (1.11;2.16)** | 511 | 0.92 (0.55;1.54) |
| **Mineral dust** | | | | | | | | |
| No | 23128 | Ref. | 23343 | Ref. | 21541 | Ref. | 8469 | Ref. |
| Low | 4266 | 0.89 (0.77;1.04) | 4331 | 0.97 (0.81;1.15) | 3916 | 0.89 (0.70;1.14) | 1526 | **0.68 (0.48;0.95)** |
| High | 1596 | 1.18 (0.96;1.45) | 1601 | 1.22 (0.97;1.54) | 1408 | 1.11 (0.79;1.57) | 562 | 1.25 (0.81;1.92) |
| **Gases and fumes** | | | | | | | | |
| No | 15896 | Ref. | 16050 | Ref. | 14803 | Ref. | 5833 | Ref. |
| Low | 11368 | 1.05 (0.93;1.18) | 11486 | 1.05 (0.92;1.20) | 10525 | 1.19 (0.99;1.44) | 4114 | 1.03 (0.83;1.28) |
| High | 1726 | 1.12 (0.91;1.38) | 1739 | 1.05 (0.82;1.33) | 1537 | 1.10 (0.77;1.56) | 610 | 1.19 (0.76;1.86) |
| **Pesticides** | | | | | | | | |
| No | 27496 | Ref. | 27766 | Ref. | 25505 | Ref. | 10065 | Ref. |
| Low | 1144 | 1.19 (1.42;2.08) | 1156 | 1.08 (0.82;1.44) | 1045 | 1.34 (0.91;1.96) | 379 | 0.81 (0.44;1.51) |
| High | 350 | 1.42 (0.96;2.08) | 353 | **1.77 (1.19;2.63)** | **315** | **2.24 (1.32;3.81)** | 113 | **2.29 (1.13;4.66)** |
| **Solvents** | | | | | | | | |
| No | 20455 | Ref. | 20691 | Ref. | 18967 | Ref. | 7452 | Ref. |
| Low | 7272 | 1.08 (0.95;1.21) | 7323 | 1.06 (0.92;1.21) | 6763 | 1.18 (0.98;1.43) | 2634 | 1.20 (0.96;1.50) |
| High | 1263 | 1.07 (0.85;1.35) | 1261 | 1.05 (0.80;1.37) | 1135 | 0.85 (0.56;1.29) | 471 | 1.17 (0.73;1.85) |
| **Metals** | | | | | | | | |
| No | 27610 | Ref. | 27888 | Ref. | 25635 | Ref. | 10032 | Ref. |
| Low | 796 | 1.01 (0.76;1.35) | 801 | 1.03 (0.74;1.41) | 721 | 1.10 (0.70;1.72) | 317 | 0.80 (0.42;1.54) |
| High | 584 | 1.16 (0.85;1.58) | 586 | 1.02 (0.71;1.46) | 509 | 0.70 (0.38;1.31) | 208 | 1.22 (0.62;2.38) |

OR=Odds ratio; CI=Confidence interval, No exposure as reference group

**Supplementary table E5: Association between biological dust and the development of respiratory symptoms and airway obstruction in subjects who were exposed only to biological dust but not to pesticides. The logistic regression was adjusted for baseline age, sex, height, education, monthly income, pack-years, and smoking.**

| Occupational exposures | N (29769) | Chronic cough OR (95%CI) | N (30097) | Chronic phlegm OR (95%CI) | N (27448) | Chronic bronchitis OR (95%CI) | N (10815) | Airway obstruction OR (95%CI) |
|---|---|---|---|---|---|---|---|---|
| **Biological dust** | | | | | | | | |
| No | 20630 | Ref. | 20845 | Ref. | 18987 | Ref. | 7572 | Ref. |
| Low | 8640 | 0.93 (0.83;1.05) | 8762 | 0.91 (0.80;1.03) | 8029 | 0.93 (0.77;1.12) | 3055 | 1.07 (0.86;1.33) |
| High | 499 | 1.16 (0.84;1.60) | 490 | 1.02 (0.70;1.48) | 432 | 1.12 (0.66;1.89) | 188 | 1.27 (0.68;1.81) |

OR=Odds ratio; CI=Confidence interval, No exposure as reference group



Faruque MO, *et al. Thorax* 2021; 76:790–797. doi: 10.1136/thoraxjnl-2020-216721
